# Effects of Epigallocatechin-3-Gallate on Autophagic Lipolysis in Adipocytes

**DOI:** 10.3390/nu9070680

**Published:** 2017-06-30

**Authors:** Sang-Nam Kim, Hyun-Jung Kwon, Seun Akindehin, Hyun Woo Jeong, Yun-Hee Lee

**Affiliations:** 1 College of Pharmacy, Yonsei Institute of Pharmaceutical Sciences, Yonsei University, Incheon 21983, Korea; sangnamik@nate.com (S.-N.K.); junek0603@gmail.com (H.-J.K.); akindehin@gmail.com (S.A.); 2 Vital Beautie Division, Amorepacific R&D Center, 314-1 Bora-dong, Giheung-gu, Yongin-si, Gyeonggi-do 17074, Korea; misterjay@amorepacific.com

**Keywords:** green tea, adipocytes, autophagy, epigallocatechin-3-gallate

## Abstract

Previous studies demonstrated effects of green tea on weight loss; however, green tea-induced modulation of adipocyte function is not fully understood. Here, we investigated effects of the major green tea phytochemical, epigallocatechin-3-gallate (EGCG) on triglyceride contents, lipolysis, mitochondrial function, and autophagy, in adipocytes differentiated from C_3_H_10_T1/2 cells and immortalized pre-adipocytes in vitro. EGCG reduced the triglycerol content significantly in adipocytes by 25%, comparable to the nutrient starvation state. EGCG did not affect protein kinase A signaling or brown adipocyte marker expression in adipocytes; however, EGCG increased autophagy, as measured by autophagy flux analysis and immunoblot analysis of LC3B, ATG7, and Beclin1. EGCG treatment reduced mitochondrial membrane potential by 56.8% and intracellular ATP levels by 49.1% compared to controls. Although mammalian target of rapamycin signaling was not upregulated by EGCG treatment, EGCG treatment induced AMP-activated protein kinase phosphorylation, indicating an energy-depleted state. In addition, EGCG increased the association between RAB7 and lipid droplets, suggesting that lipophagy was activated. Finally, knockdown of *Rab7* attenuated the EGCG-dependent reduction in lipid contents. Collectively, these results indicated that EGCG upregulated autophagic lipolysis in adipocytes, supporting the therapeutic potential of EGCG as a caloric restriction mimetic to prevent obesity and obesity-related metabolic diseases.

## 1. Introduction

Green tea is consumed worldwide, and has been shown to have various health benefits [1], including effects on weight loss and metabolic health improvement [2,3] The major bioactive component in green tea is a polyphenolic catechin, epigallocatechin-3 gallate (EGCG) [1], to which the weight loss effects of green tea have been attributed [2]. Although obesity is clearly associated with metabolic syndrome [4], excessive fat mass per se does not appear to be the cause since lipodystrophic patients, who lack adequate fat mass, are also highly insulin resistant [4,5]. Rather, disease occurs when the functions of adipose tissues in metabolic homeostasis are impaired [4].

Adipocytes are a specialized cell type that can store energy in the form of neutral lipids, mainly triglycerides (TGs) [4]. In addition to this anabolic function, breakdown of TGs occur in adipocytes, contributing to mobilization of fatty acids from adipose tissues into circulation and other tissues [6]. Lipolysis can be defined as the hydrolysis of TGs into glycerol and fatty acids, and the three main lipolytic enzymes involved in TG hydrolysis have been identified, namely adipose triglyceride lipase (ATGL), hormone-sensitive lipase (HSL), and monoacylglycerol lipase (MGL) [7]. Lipolysis in adipocytes is controlled primarily by β-adrenergic stimulation [6], which mediates cAMP-dependent protein kinase A (PKA) downstream signaling [7]. In particular, phosphorylation of HSL by PKA increases the enzyme activity and translocation from the cytosol into lipid droplets [7]. Thus, understanding of impact of EGCG on adipocyte lipolysis is important to decipher the molecular mechanisms involved in the beneficial effects of green tea in metabolic health. However, the effects of green tea on adipocyte lipid metabolism have not yet been investigated.

In addition to lipolysis by cytosolic lipases, as mentioned above, autophagy of lipid droplets has been recognized as a complementary pathway for cellular lipid breakdown [8]. Autophagy is a process through which cells consume themselves and is induced by nutrient starvation, calorie restriction, and potential calorie restriction mimetics (CRMs) [9]. Recently, induction of autophagy by EGCG has been investigated in several cell types, including hepatocytes and vascular cells [8]; however, the effects of EGCG on autophagic lipolysis in adipocytes have not been examined.

To investigate the regulatory roles of EGCG in adipocyte lipid metabolism, we examined the effect of EGCG on the metabolic functions of adipocytes differentiated from C_3_H_10_T1/2 [10], 3T3-L1, and immortalized pre-adipocytes. We then investigated the role of EGCG in lipid catabolism pathways, including adrenergic activation of lipolysis, induction of brown adipocyte phenotypes, and autophagy-related degradation of lipid droplets in adipocytes. To further investigate the mechanisms by which EGCG reduces lipid contents in adipocytes, the mammalian target of rapamycin (mTOR) and AMP-activated protein kinase (AMPK) signaling pathways were analyzed. Finally, the involvement of autophagic lipolysis in the activation of lipid catabolism by EGCG was investigated by knockdown of *RAB7*, a lipophagy-related gene in adipocytes.

## 2. Materials and Methods

### 2.1. Cell Cultures

C_3_H_10_T1/2 cells and 3T3-L1 preadipocytes were obtained from ATCC (Manassas, VA, USA) and cultured, as previously described [11]. Briefly, cells were cultured to confluence in growth medium (Dulbecco’s modified Eagle’s medium (DMEM: Sigma, St. Louis, MO, USA) supplemented with 10% fetal bovine serum (FBS, Gibco, Thermo Fisher Scientific, Waltham, MA, USA) and 1% penicillin/streptomycin (Welgene, Gyeongsan-s, Gyongsangbuk-do, Korea) at 37 °C in a humidified atmosphere with 5% CO_2_, and then exposed to adipogenic differentiation medium (DMEM containing BMP4 (20 ng/mL, R&D systems, Minneapolis, MN, USA), 0.125 mM indomethacin (Cayman, Ann Arbor, MI, USA), 2.5 mM isobutylmethylxanthine (IBMX, Cayman, Ann Arbor, MI, USA), 1 uM dexamethasone (Cayman, Ann Arbor, MI, USA), 10 ug/mL insulin (Sigma, St. Louis, MO, USA) and 1 nM triiodothyronine (T3, Cayman, Ann Arbor, MI, USA) for three days. For maintenance of adipogenic differentiation, cells were exposed to DMEM containing 10% FBS, 10 μg/mL insulin (Sigma, St. Louis, MO, USA) and 1 nM triiodothyronine (T3, Cayman, Ann Arbor, MI, USA) for three days.

To prepare immortalized preadipocytes with the potential to become brown adipocytes, interscapular brown adipose tissues were isolated from C57BL/6 mice [12]. Primary preadipocytes were collected in stromovascular factions by collagenase digestion and centrifugation, as previously described [12]. For retrovirus production, viral constructs for SV40 large and small T antigens (plasmid #13970, Addgene, Cambridge, MA, USA) were transfected into phoenix cells using Lipofectamin 2000. Immortalized preadipocytes are expanded in growth medium (DMEM with 10% FBS and 1% penicillin/streptomycin) and differentiated in adipogenic differentiation medium for three days. Cells were maintained in medium containing insulin for up to two weeks.

Fully differentiated adipocytes were exposed to DMEM supplemented with 10% FBS overnight and then treated with indicated concentration of EGCG (purity >95%; Sigma, St. Louis, MO, USA) or quercetin (purity >95%; Sigma, St. Louis, MO, USA). Earle’s balanced Salt Solution (EBSS, Thermo Fisher, Waltham, MA, USA) was used for nutrient starvation. 8-bromoadenosine 3′5′-cyclic monophosphate (8-Br-cAMP) was used for PKA activation.

For inhibition of autophagy, chloroquine (an inhibitor of late phase (lysosomal degradation), 50 μM, Sigma, St. Louis, MO, USA) and 3-methyl adenine (3-MA, an inhibitor of early phase, 10 mM, Sigma, St. Louis, MO, USA) were used. Adipocytes were pretreated with inhibitors for 30 min before EGCG treatment.

For Rab7 knockdown, siRNA targeting Rab7 (cat. no. #EMU150241; Sigma, St. Louis, MO, USA) was transfected into adipocytes differentiated from C_3_H_10_T1/2 cells, using Lipofectamin2000 (Thermo Fisher, Waltham, MA, USA). Intracellular TG content was determined using a commercially available triglyceride colorimetric assay kit (Cayman Chemicals, Ann Arbor, MI, USA). ATP levels were measured with an ATP Assay Kit (Roche, Indianapolis, IN, USA). For the assays, C_3_H_10_T1/2 cells were plated on 12-well plates at a cell density of 10^5^ cells/mL and differentiated into adipocytes, as described above. Cell lysates were prepared in each sample buffer from the assay kit. 10 μL of cell lysate (4 × 10^4^ cells/assay) was used for analysis of TG contents, and 25 μL of cell lysate (10^5^ cells/assay) was used for analysis of ATP levels. Data were normalized based on the protein concentration of the corresponding cell lysate.

### 2.2. Gene Expression Analysis

RNA was extracted using the TRIzol^®^ reagent (Invitrogen, Carlsbad, CA, USA), and 1 μg of RNA was reverse transcribed using a cDNA synthesis kit (High-capacity cDNA Reverse Transcription kit; Applied Biosystems, Foster City, CA, USA). One hundred nanograms of cDNA was subjected to quantitative polymerase chain reaction (qPCR) in 20-μL reaction volumes (iQ SYBR Green Supermix; Bio-Rad, Hercules, CA, USA) with 100 nM primers. qRT-PCR was performed using SYBR Green dye and CFX Connect Real-time system (Bio-Rad, Hercules, CA, USA) for 45 cycles and fold change for all samples was calculated by using the 2^−^ΔΔCt method. Peptidylprolyl Isomerase A (PPIA) was used as a housekeeping gene for mRNA expression analysis. Primers used for qRT-PCR were described previously [11].

### 2.3. Western Blotting

Protein were extracted in RIPA buffer (Thermo Fisher, Waltham, MA, USA) containing protease (Sigma, St. Louis, MO, USA) and phosphatase Roche, Indianapolis, IN, USA) inhibitors, as previously described [12]. Resolved proteins were transferred to polyvinylidene difluoride (PVDF) membranes, and membranes were blocked for 1 h at room temperature in 5% bovine serum albumin or 5% powdered skim milk in TBST. Then, the membranes were incubated with primary antibodies overnight at 4 °C. Blots were then washed, incubated with a secondary anti-rabbit horseradish peroxidase antibody (diluted 1:5000 in TBST; Cell Signaling Technology, Danvers, MA, USA) for 30 min at room temperature, and visualized with SuperSignal West Dura Substrate (Pierce-Invitrogen, Waltham, MA, USA). The following primary antibodies were used for Western blot analysis: anti-UCP1 (rabbit, Alpha Diagnostic International, San Antonio, TX, USA), anti-cytochrome c oxidase subunit IV (COX IV; rabbit, Cell Signaling, Danvers, MA, USA), phospho-HSL (Ser563, rabbit, Cell Signaling, Danvers, MA, USA), HSL (rabbit, Cell Signaling, Danvers, MA, USA), LC3B (rabbit, Cell Signaling, Danvers, MA, USA), anti-cAMP responsive element binding protein (CREB; rabbit, Cell Signaling, Danvers, MA, USA), phospho-CREB (Ser133, rabbit, Cell Signaling, Danvers, MA, USA), AMPK (rabbit, Cell signaling, Danvers, MA, USA), phospho-AMPK (Thr172, rabbit, Cell Signaling, Danvers, MA, USA), mTOR (rabbit, Cell Signaling, Danvers, MA, USA), phospho-mTOR (Ser2481, rabbit, Cell Signaling, Danvers, MA, USA), PLIN1 (rabbit, Santacruz, Dallas, TA, USA), RAB7 (rabbit, Cell Signaling, Danvers, MA, USA), anti-RAB7 (rabbit, Cell Signaling), anti-ATG7 (rabbit, Cell Signaling, Danvers, MA, USA), anti-BECLIN1 (rabbit, Cell Signaling, Danvers, MA, USA), and a/b tubulin (rabbit, Cell Signaling, Danvers, MA, USA).

### 2.4. Analysis of Autophagic Flux

For autophagic flux analysis, C_3_H_10_T1/2 cells were infected with pBABE-puro mCherry-EGFP-LC3B [13] (Plasmid #22418, Addgene, Cambridge, MA, USA, a gift from Jayanta Debnath) by using retrovirus infection as described previously. Autophagic flux was measured by quantifying the pH-sensitive decrease in green fluorescent protein (GFP) intensity over red fluorescent protein (RFP) intensity as an indication of autolysosome formation. For autophagic flux analysis by flow cytometry, analytic cytometry was performed using BD FACSAria III (BD Biosciences, San Jose, CA, USA). Raw data were processed using FlowJo software (Tree Star, Ashland, OR, USA). Alternatively, long-term live-cell imaging was performed every 1 h with IncuCyte ZOOM Live Cell Imaging equipment (Essen Bioscience, Ann Arbor, MI, USA), and the green and red fluorescence intensities of the images were analyzed using ImageJ (imagej.nih.gov, accessed on 1/22/2016).

### 2.5. Analysis of Mitochondrial Function

To measure mitochondrial membrane potential, adipocytes cultured in 24-well plates (10^5^ cells/well) were incubated with 0.4 μM JC-1 (Sigma, St. Louis, MO, USA) for 30 min. The fluorescence signal was determined by using Tecan microplate reader at 485 nm excitation and 527 nm emission for green fluorescence, and 485 nm excitation and 590 nm emission for red fluorescence. To measure oxygen consumption, adipocytes differentiated from C_3_H_10_T1/2 cells (5 × 10^6^ cells/assay) were collected in a hypotonic medium containing 120 mM KCl, 5 mM KH_2_PO_4_, 3 mM HEPES, 1 mM EGTA and 1 mM MgCl_2_ (pH 7.2) at 35 °C. Succinate (10 mM), digitonin (4 μg/mL), adenosine diphosphate (ADP, 1 mM), oligomycinA (0.2 uM), carbonyl cyanide-4-(trifluoromethoxy) phenylhydrazone (FCCP, 0.5 μM) and KCN (0.1 mM) were added sequentially. Oxygen concentrations and oxygen consumption rates were measured by the Oxygraph plus system (Hansatech, Norfolk, UK) with chart recording software. OCRs were normalized according to protein concentrations. Uncoupled respiration was calculated by subtraction of the KCN-induced OCR from the oligomycin A-induced OCR. ATP related respiration was calculated by subtraction of the oligomycin A-induced OCR from the basal OCR.

### 2.6. Immunofluorescence Staining

For immunofluorescence staining, cells were cultured in four-chamber cell culture slides (SPL), fixed with paraformaldehyde (4% in phosphate-buffered saline (PBS)) and subjected to immunocytochemical analysis as previously described [14]. Briefly, fixed cells were incubated with blocking buffer (5% normal goat serum in PBS) and permeabilization buffer (0.5% TritonX100 in PBS) for 30 min at room temperature. The slides were incubated with a primary antibody in blocking buffer overnight at 4 °C, washed, and then incubated with a secondary antibody in blocking buffer for 1 h at room temperature. Antibodies used for immunofluorescence detection were anti-RAB7 antibody (rabbit, 1:100, Cell Signaling, Danvers, MA, USA), PLIN2 (mouse, 1:100, Santacruz, Dallas, TA, USA), and LC3 (rabbit, 1:100, Cell signaling, Danvers, MA, USA). The secondary antibodies used were goat anti-rabbit-Alexa Fluor 488 and goat anti-mouse-Alexa Fluor 594 (1:500, Invitrogen, Carlsbad, CA, USA). For the negative control, primary antibodies were omitted. DAPI (Sigma) was used for nuclear counterstaining. Intracellular neutral lipid was stained with BODIPY^®^ 493/503 (4,4-Difluoro-1,3,5,7,8-Pentamethyl-4-Bora-3a,4a-Diaza-s-Indacene, Thermo Fisher, Waltham, MA, USA) or HCS LipidTox Deep Red Neutral Lipid stain, for cellular imaging.

### 2.7. Statistical Analysis

Statistical analyses were performed using GraphPad Prism 5 software (GraphPad Software, La Jolla, CA, USA). Data are presented as mean ± standard errors of the means (SEMs). Statistical significance between two groups was determined by unpaired *t*-test, as appropriate. Comparisons among multiple groups was performed using a one-way or two-way analysis of variance (ANOVA), with Bonferroni post hoc tests to determine *p* values.

## 3. Results

### 3.1. EGCG Reduced Lipid Content in Adipocytes

**Figure 1:**
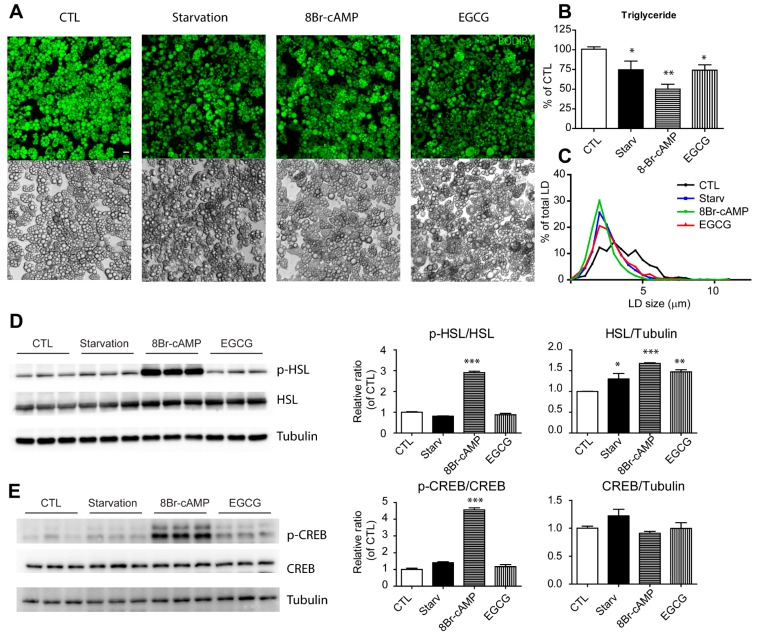
Effects of EGCG on lipid content and PKA signaling in adipocytes (**A**–**C**) BODIPY staining (**A**), intracellular triglyceride levels (**B**), dimeters of lipid droplets (**C**) in adipocyte differentiated from C_3_H_10_T1/2 cells treated with starvation medium, 8Br-cAMP (1 mM) and EGCG (10 μM) for 24 h (**D**,**E**) Immunoblot analysis of p-HSL, HSL, p-CREB, and CREB in adipocyte differentiated from C_3_H_10_T1/2 cells treated with starvation medium, 8Br-cAMP (1 mM) and EGCG (10 μM) for 24 h. *p* values were calculated using the two-tailed unpaired *t*-test (*n* = 3, means ± SE; * *p* < 0.05, ** *p* < 0.01, *** *p* < 0.001).

To test the effect of EGCG on lipid contents, differentiated adipocytes from C_3_H_10_T1/2 cells were treated with EGCG under conditions that activate lipolysis (Figure 1). EGCG was used at concentration of 10 μM according to previous works [13]. Then 8Br-cAMP was used as a positive control that activates PKA and downstream events, including lipolysis. In addition, we included nutrient starvation medium. As expected, nutrient starvation and 8Br-cAMP treatment reduced neutral lipid contents significantly, as measured by BODIPY staining in live cells (Figure 1A) and reduced their intracellular TG contents (Figure 1B). EGCG reduced the neutral lipid TG content significantly, and this reduction was comparable with the levels in the nutrient starvation state (Figure 1A,B). The size of the lipid droplets (LDs) was also reduced in adipocytes treated with EGCG (Figure 1C: mean diameters of LDs: control (CTL) = 3.59 μm; EGCG = 2.72 μm; starvation = 2.62 μm; cAMP = 2.22 μm). PKA signaling is a well-known pathway that activates lipolysis in adipocytes [7,14]; therefore, we examined phosphorylation of the PKA downstream target proteins that are involved in upregulation of lipolysis: hormone sensitive lipase (HSL) and cAMP response element-binding protein (CREB). HSL is a primary lipolytic enzyme responsible for hydrolysis of diacylglycerol in adipocytes [7]. CREB is a transcription factor that can be activated by phosphorylation of the Ser133 residue by PKA activation and upregulates the expression of HSL as a downstream target [15]. We found that 8Br-cAMP increased the phosphorylation levels of HSL and CREB significantly (by three-fold and four-fold, respectively) compared with control conditions (Figure 1D,E). However, neither starvation nor EGCG treatment increased the phosphorylation levels. Interestingly, expression of HSL was significantly higher in the EGCG-treated groups than in the controls. The data suggested that EGCG reduced the lipid contents effectively in adipocytes; however, this phenomenon is independent of PKA signaling.

### 3.2. Effects of EGCG on Browning of Adipocytes

**Figure 2:**
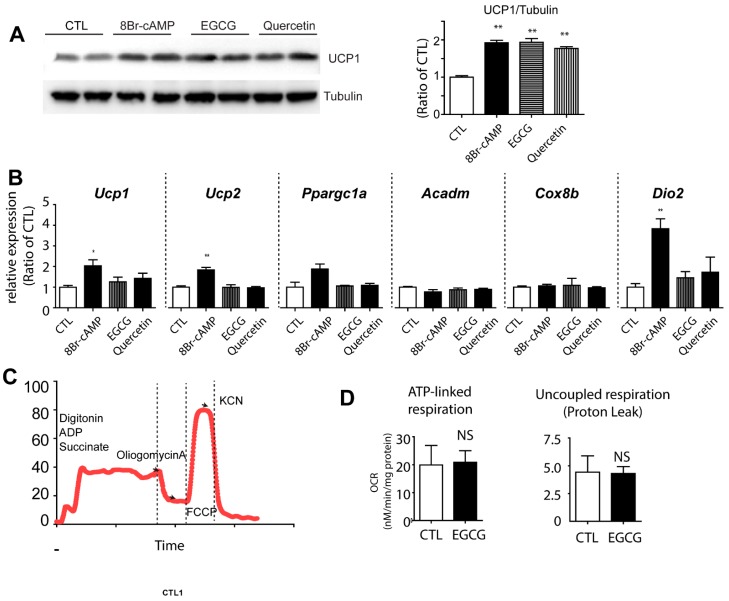
Effects of EGCG on brown adipocyte marker expression in adipocyte cultures. Immunoblot analysis of UCP1 expression (**A**) and quantitative PCR analysis (**B**) of genes involved in mitochondrial metabolism and brown adipocyte markers in adipocytes differentiated from C_3_H_10_T1/2 treated with 8Br-cAMP (1 mM), EGCG (10 μM) and quercetin (10 μM) for 24 h (**C**,**D**) Analysis of oxygen consumption of adipocytes differentiated from C_3_H_10_T1/2 cells treated with EGCG for 24 h; (**C**) An example of analysis of oxygen consumption rate (OCR) with a series of treatments of indicated drugs; (**D**) Comparisons of ATP-linked respiration and uncoupled respiration between cells treated with EGCG and vehicle controls. *p* values were calculated using the two-tailed unpaired *t*-test (*n* = 3, means ± SE; * *p* < 0.05, ** *p* < 0.01).

Non-shivering thermogenesis via uncoupling protein 1 (UCP1) expression in brown adipocytes is one of the mechanisms that activates catabolic metabolism to reduce the lipid content. Although it has been suggested that in vivo thermogenesis can be stimulated by green tea extract treatment (by upregulation of sympathetic tones) [16], the direct effects of EGCG on browning of white adipocytes has not been investigated. Therefore, we evaluated the effects of EGCG on browning of adipocytes differentiated from C_3_H_10_T1/2 cells (Figure 2A,B). In addition to EGCG, quercetin was tested because its browning effect has been reported previously [17].

EGCG treatment upregulated the UCP1 protein level by 2 fold (Figure 2A). However, the transcript levels of *UCP1* were not significantly induced by EGCG treatment (Figure 2B). In addition, Ucp1 mRNA levels were 300,000-fold lower than those in in vivo brown adipocytes (i.e., C_3_H_10_T1/2 *Ucp1* (% of *PPIA*) 0.01 ± 0.005 vs. BAT *Ucp1* (% of *PPIA*) = 3287 ± 1100, *n* = 6). Similarly, EGCG did not increase a brown adipocyte marker (*Dio2*) and genes involved in mitochondrial metabolism (i.e., *Ppargc1a, Acdam, Cox8b, Ucp2*) expression in adipocytes differentiated from C_3_H_10_T1/2 cells (Figure 2B). Analysis of oxygen consumption rate demonstrated that EGCG treatment did not affect either ATP-linked respiration or uncoupled respiration (Figure 2C,D). This suggested that upregulation of UCP1 expression or mitochondrial respiration does not seem to be a major contributor to the loss of lipids.

**Figure 3:**
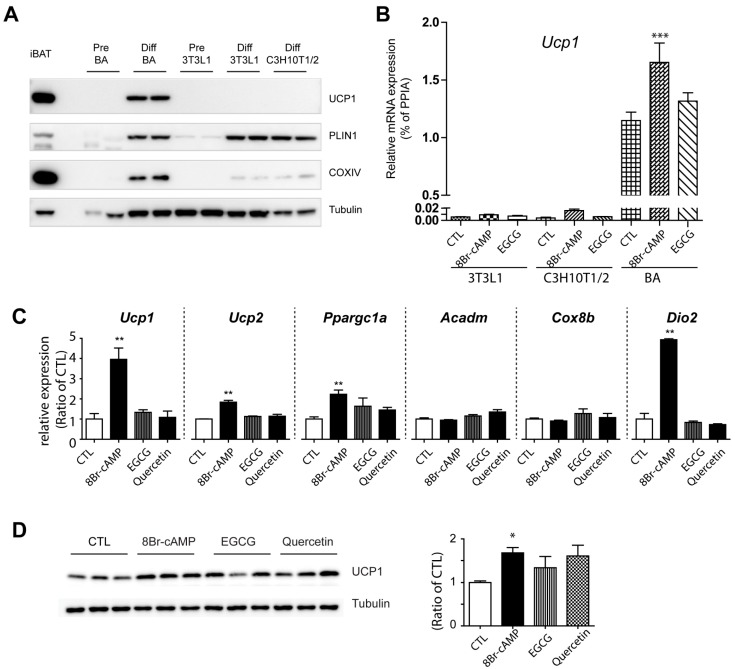

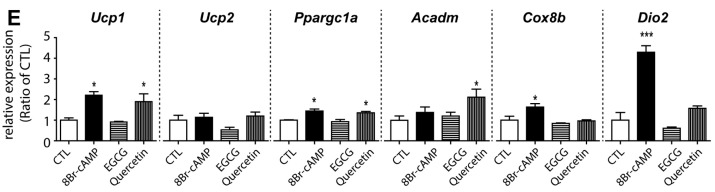
Establishment of BA cell lines and effects of EGCG on brown adipocyte marker expression in adipocyte cultures. Immunoblot analysis of UCP1, perilipin1 (PLIN1), and COXIV expression (**A**) and quantitative PCR analysis of UCP1 expression (**B**) in adipocytes differentiated from 3T3L1, C_3_H_10_T1/2, and immortalized preadipocytes isolated from iBAT. Two-way ANOVA revealed significant main effects of treatment (*p* = 0.0165) and cell types (*p* < 0.0001) in UCP1 expression and significant interaction of treatment and cell types (*p* = 0.0060). Significant differences between control and treated group were determined by post-hoc pairwise comparison with Bonferroni correction (mean ± SEM; *n* = 4 per condition, ***p < 0.001). (**C**) Quantitative PCR analysis of brown adipocyte gene expression in adipocytes differentiated from 3T3L1 treated with 8Br-cAMP (1 mM), EGCG (10 μM) and quercetin (10 μM) for 24 h. Immunoblot analysis of UCP1 expression (**D**) and quantitative PCR analysis (**E**) of brown adipocyte gene expression in adipocytes differentiated from BA cell lines treated with 8Br-cAMP (1 mM), EGCG (10 μM) and quercetin (10 μM) for 24 h. *p* values were calculated using the two-tailed unpaired *t*-test (*n* = 3, means ± SE; * *p* < 0.05, ** *p* < 0.01, *** *p* < 0.001).

To test whether the minimal effect of UCP1 expression in adipocytes is related to cell type-specific gene expression machinery, we tested the effect of EGCG on brown adipocyte cell lines and 3T3L1 cells. We established brown adipocyte cell lines by immortalization of preadipocytes isolated from mouse interscapular BAT. As shown in Figure 3A, expression of UCP1 protein was confirmed in adipocytes differentiated from immortalized brown preadipocytes (Figure 3A). Ucp1 mRNA expression was approximately 1000 fold higher in the brown adipocytes, compared to the levels in 3T3L1 and C_3_H_10_T1/2 (Figure 3B). The gene expression patterns in adipocytes differentiated from 3T3L1 were similar to adipocytes from C_3_H_10_T1/2 cells resembling white adipocyte phenotypes (Figure 2B,C). In addition, EGCG did not increase Ucp1 expression and other genes involved in mitochondrial functions in brown adipocytes significantly (Figure 2D,E), which indicated that EGCG is not a thermogenic signal for the activation of brown adipocyte metabolism.

### 3.3. Effect of EGCG on Autophagy

**Figure 4:**
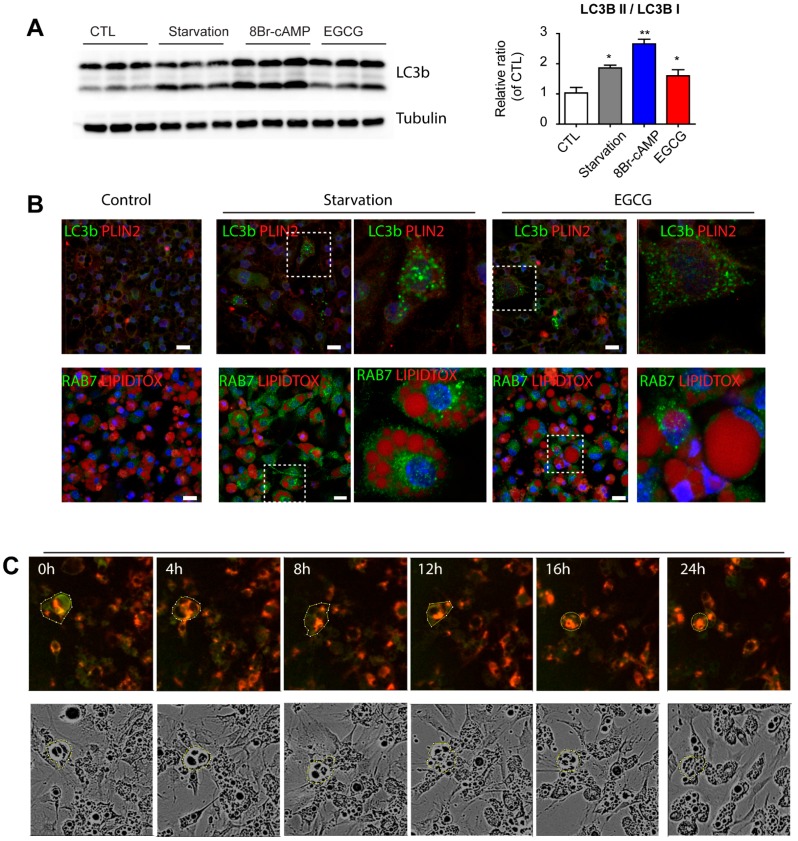

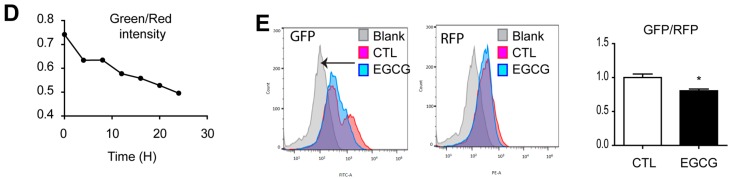
Effects of EGCG on autophagy responses in adipocytes. (**A**) Immunoblot analysis of LC3b expression in adipocytes differentiated from C_3_H_10_T1/2 treated with starvation medium, 8Br-cAMP (1 mM) and EGCG (10 μM) for 1 day. *p* values were calculated using the two-tailed unpaired *t*-test (*n* = 3, means ± SE; * *p* < 0.05, ** *p* < 0.01); (**B**) Immunofluorescence staining of LC3b/PLIN2 or RAB7/LipidTox in adipocytes differentiated from C_3_H_10_T1/2 treated with 8Br-cAMP (1 mM) and EGCG (10 μM) for 24 h. Nuclei were counterstained with DAPI. Bars = 20 μm; (**C**) Representative images from long-term live imaging of adipocytes expressing LC3B-GFP-RFP reporters treated with EGCG; (**D**) Time course analysis of GFP/RFP ratio from (**C**). (**E**) Flow cytometric analysis of adipocytes expressing LC3B-GPF-RFP reporters treated with ECGC.

**Figure 5:**
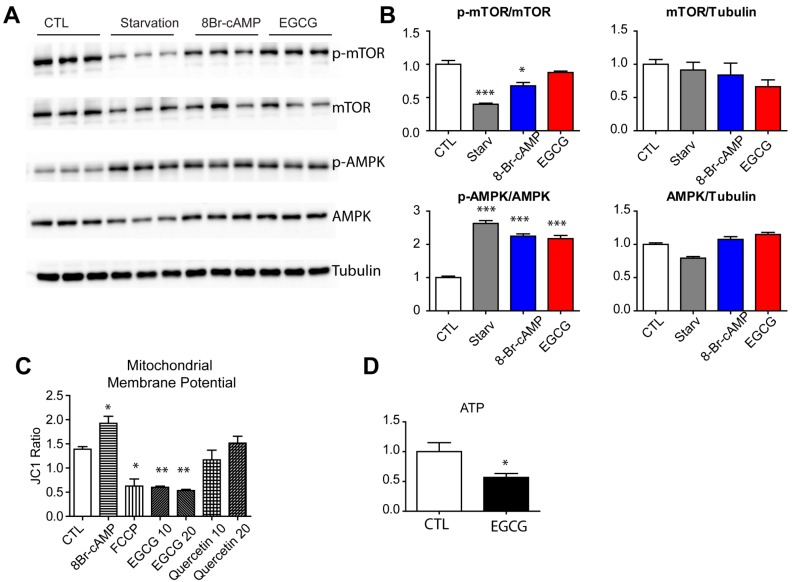
Effects of EGCG on activation of mTOR and AMPK in adipocytes. (**A**,**B**) Immunoblot analysis of phosphorylation of mTOR and AMPK in adipocytes differentiated from C_3_H_10_T1/2 treated with starvation medium, 8Br-cAMP (1 mM) and EGCG (10 μM) for 24 h. Mitochondrial membrane potential (**C**), and intracellular ATP (**D**) in adipocytes differentiated from C_3_H_10_T1/2 treated with 8Br-cAMP (1 mM), FCCP (0.5 μM), EGCG (10 or 20 μM) or quercetin (10 or 20 μM) for 2 h. *p* values were calculated using the two-tailed unpaired *t*-test (*n* = 3, means ± SE; * *p* < 0.05, ** *p* < 0.01, *** *p* < 0.001).

EGCG reduced the lipid content in adipocytes without activation of HSL and PKA signaling; thus, we hypothesized that EGCG activates an alternative pathway to consume lipids. Autophagy has been reported as a novel regulator of lipolysis [8], acting as a lysosomal lipolytic pathway; therefore, we examined autophagic responses after EGCG treatment. Differentiated adipocytes from C_3_H_10_T1/2 cells were grown in nutrient starvation medium, and standard growth medium containing EGCG or 8-Br-cAMP. To measure autophagic flux, conversion of LC3B-I into LC3B-II was determined by Western blotting analysis. As shown in Figure 4A, an increased ratio of LC3B-II to LC3B-I was observed in all three conditions, including EGCG and 8Br-cAMP treatment. Interestingly, 8Br-cAMP treatment (i.e., PKA activation) increased LC3B-II generation to levels similar to that induced by starvation (Figure 4A). EGCG treatment increased other autophagy markers, Beclin1 and ATG7 expression (Figure S1). Treatment with chloroquine, an inhibitor of lysosomal degradation, increased accumulation of LC3bII in control and EGCG-treated groups, while 3-MA reduced LC3BII. In agreement with the immunoblotting analysis, immunofluorescence staining of adipocytes treated with EGCG showed punctuated patterns of LC3B staining (Figure 4B), which is a prominent feature of autophagy. In addition, EGCG increased the association between RAB7 and lipid droplets, indicating activation of lipophagy (Figure 4B). Next, autophagic flux was measured using LC3 tandemly tagged with fluorescent proteins that detect lysosomal degradation [18] in C_3_H_10_T1/2 cells. This system expresses a chimeric LC3 fused with eGFP and mCherry; thus, the pH-sensitive decrease in GFP intensity over RFP intensity indicates autolysosome formation. As shown in Figure 4C, long-term live cell imaging demonstrated that EGCG treatment induced a decrease in the green and red fluorescence intensity ratio during the course of the treatment (Figure 5C,D). Furthermore, flow cytometry analysis confirmed the decrease in green fluorescent intensity by EGCG treatment (Figure 3E). Collectively, analyses of the autophagic response supported the induction of autophagy by EGCG.

Autophagy can be activated by various extracellular and intracellular stimuli. Importantly, starvation can lead to inhibition of the mammalian target of rapamycin (mTOR) pathway, which is known as a nutrient sensing signaling pathway. To test whether EGCG mimics starvation by inhibiting of mTOR signaling, we examined the phosphorylation levels of mTOR using Western blotting. As shown in Figure 5A, starvation conditions and 8Br-cAMP treatment reduced mTOR phosphorylation significantly; however, EGCG treatment did not affect p-mTOR or total mTOR levels. AMP-activated protein kinase (AMPK) can directly phosphorylate UNC-51-Like kinase 1 (ULK1), a key regulator of autophagy induction during energy starvation; thus, we examined p-AMPK levels [15]. Starvation, 8Br-cAMP, and EGCG treatment increased the phosphorylation of AMPK, indicating depleted intracellular energy levels. Mitochondrial oxidative phosphorylation is the major pathway of ATP generation; thus, we examined the mitochondrial membrane potential. The data indicated that the membrane potential was reduced by EGCG treatment while it was increased by 8Br-cAMP treatment (Figure 5C). Consistent with this signaling status, EGCG treatment reduced intracellular ATP levels in adipocytes (Figure 5D).

**Figure 6:**
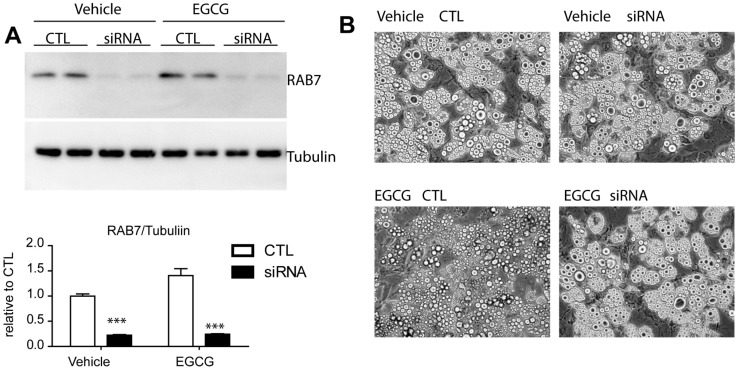
Effects of knockdown of RAB7 on EGCG induced reduction in lipid content in adipocytes. (**A**) Immunoblot analysis of RAB7 in adipocytes differentiated from C_3_H_10_T1/2 treated with siRNA or scrambled sequence controls. Two-way ANOVA revealed significant main effects of siRNA knockdown (*p* < 0.0001) and EGCG treatment (*p* = 0.0122) in RAB7 expression and significant interaction of siRNA knockdown and EGCG treatment (*p* = 0.0206). Significant differences between control and siRNA were determined by post-hoc pairwise comparison with Bonferroni correction (mean ± SEM; *n* = 4 per condition, *** *p* < 0.001) (**B**) Representative images of adipocytes differentiated from C_3_H_10_T1/2 treated with vehicle or EGCG (10 μM) for 24 h. (*n* = 4, means ± SE; *** *p* < 0.001).

Lipophagy has been described as a form of autophagy that is specialized for the degradation of lipid droplets, and RAB7 was reported as a key molecule that enables the specific recognition of LD proteins in the process of lipophagy [19]. Autophagy related genes, including *Rab7*, could affect the differentiation of adipocytes [20]; therefore, we silenced *Rab7* to transiently knockdown its expression using a siRNA after full differentiation. siRNA transfection knocked down *Rab7* expression by 45% compared with negative controls using a scrambled sequence (Figure 6A). As indicated in Figure 6B, *Rab7* knockdown attenuated the reduction in the lipid content induced by EGCG treatment in adipocytes.

## 4. Discussion

Metabolic disease is a multifactorial disease and the numerous health benefits of green tea, including its weight loss effects, have been proposed to have application in the prevention and/or treatment of obesity-related metabolic disease [21]. Among the various bioactive constituents in green tea, the polyphenolic catechin EGCG has been proposed to exert a weight loss effect [2,3,21]; however, the regulatory role of EGCG in adipocyte lipid metabolism remains unclear. In this study, we examined the direct effects of EGCG on adipocyte metabolism using in vitro adipocyte cultures, including its effects on lipolysis, thermogenic marker expression, mitochondrial metabolism, and autophagy. We demonstrated that EGCG reduced lipid contents by activating autophagy, partly via a reduction in intracellular ATP levels, mimicking the energy-depleted state.

The central finding in the current study was the demonstration of EGCG-induced autophagic lipolysis in adipocytes. Although this is not the first study to report the effects of EGCG on adipocyte function; previous works have focused on the inhibitory effects of EGCG on adipogenesis [22,23] but not on adipocyte lipid catabolism. The involvement of lipophagy in the clearance of lipid droplets has been investigated in several cell types, including hepatocytes and vascular cells [8]; however, the involvement of EGCG in the direct activation of lipophagy in adipocytes has not been investigated. The in vivo effects of EGCG on the autophagic lipolysis of adipose tissue were not addressed in this study, and further studies are required. In support of the effects of EGCG on weight loss, in vivo effects of green tea in mouse models have been shown to be related to the role of EGCG in the upregulation of sympathetic nervous system activity, leading to increased energy consumption, thermogenesis, and fat oxidation [21].

In this study, we found that lipophagy was an alternative pathway to reduce lipid contents without downstream activation of PKA and that EGCG could activate this process. Our findings also suggested that lipophagy activation in adipocytes could be involved in mediating the weight loss effects of EGCG. According to a previous pharmacokinetic analysis of green tea [24], the peak concentration of EGCG in the blood reaches 326 ng/mL after a single dose (4.5 g) of green tea consumption. Considering the high concentration of EGCG (10 μM) used in this study, further studies are required for the extrapolation of in vitro data to the in vivo situation, such as dose-response curves with lower doses and analysis of the tissue distribution of EGCG. Such studies will allow us to determine whether induction of autophagic lipolysis by EGCG can be achieved by green tea consumption or pharmacologic treatment with EGCG.

Under fasting conditions, stimulation of catecholamine release into systemic circulation is considered a major catabolic signal promoting the increase of lipid mobilization from adipocytes. In this study, we examined the effects of nutrient starvation on adipocyte lipid metabolism separately from catecholamine-stimulated β-adrenergic signaling. As expected, nutrient starvation increased autophagy and lipophagy to reduce the lipid content, without catecholamine-stimulated lipolysis. These data supported the view that the response of adipocytes to nutrient scarcity is integrated and coordinated at the organismal level via intrinsic cell properties and hormonal signals. Interestingly, the cAMP analog increased the levels of autophagy, and upregulation of mTOR signaling, and AMPK activation stimulated the autophagic response. Although not investigated in this study, determining the mechanism of PKA signaling in the induction of autophagy would be informative for subsequent studies of adipocyte metabolism.

The mechanisms of autophagy induction have been investigated intensively in various research areas related to chronic disease. In this study, we showed that reduction of ATP synthesis was the major initiating stimulus for autophagy induction by EGCG. Consistent with our observations, previous studies have reported that ATP synthase activity could be inhibited by resveratrol, several antioxidant flavonoids, and polyphenolic catechins, including EGCG [25].

The development of CRM is a promising approach to prevent aging and obesity-related metabolic diseases. In this regard, natural/pharmacological autophagy inducers have been investigated as CRMs that share molecular mechanisms of health improvement with calorie restriction [9]. In this regard, EGCG has been recognized as a CRM with acetyltransferase inhibitor activity [26]. Our findings demonstrated that EGCG induced autophagic lipolysis to reduce the lipid contents in adipocytes by inhibiting mitochondrial oxidative phosphorylation, and further supported the beneficial effects of EGCG as a CRM. Our results suggested that the adipocyte-specific effects of CRMs deserve further investigation as more favorable therapeutic targets for the reduction of adipocyte mass. Furthermore, investigation of the mechanisms involved in the specific lysosomal degradation of lipid droplets in adipocytes by other CRMs would lead to the identification of novel targets for drug discovery and CRMs.

## Supplementary Materials

The following are available online at www.mdpi.com/2072-6643/9/7/680/s1, Figure S1: Effects of EGCG on autophagy responses in adipocytes.
