# Supplementary material for: Effects of Epigallocatechin-3-Gallate on Autophagic Lipolysis in Adipocytes
Source: Nutrients. 2017 Jun 30;9(7):680. doi: 10.3390/nu9070680 (PMC5537795; doi:10.3390/nu9070680)
Supplement: Supplementary file 1 [file nutrients-09-00680-s001.zip › nutrients-200943-supplementary.pdf]

Supplemental information

Article

Effects of epigallocatechin-3-gallate on autophagic lipolysis in adipocytes

Sang-Nam Kim [1#], Hyun-Jung Kwon [1#], Seun Akindehin[1], Hyun Woo Jeong, and Yun-Hee Lee [1,*]

[1] College of Pharmacy, Yonsei Institute of Pharmaceutical Sciences, Yonsei University, Incheon, Korea; sangnamik@nate.com (S.N.K.); junek0603@gmail.com (H.J.K.); akindehin@gmail.com (S.A.)

[2] Vital Beauty Division, Amorepacific R&D Center, 314-1 Bora-dong, Giheung-gu, Yongin-si, Gyeonggi-do: misterjay@amorepacific.com

[*] Correspondence: yunhee.lee@yonsei.ac.kr; Tel.: +82-32-749-4522

#**These authors contributed equally to this work**

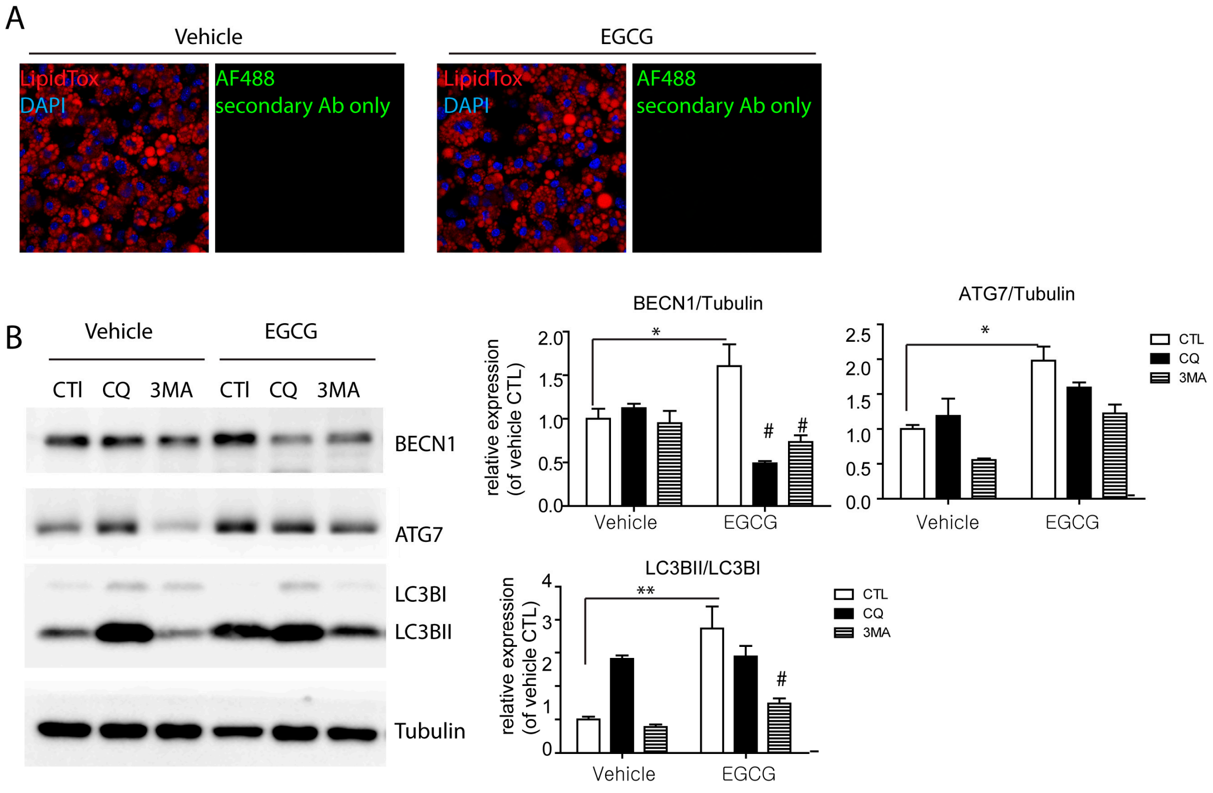

Figure S1. Effects of EGCG on autophagy responses in adipocytes (related to Figure 4).

A. Representative images of negative controls used for RAB7 and LC3B detection. The negative controls were incubated with a secondary antibody conjugated with Alexa Flour 488 without primary antibody incubation

B. Western blot analysis of beclin1, ATG7, and LC3B expression in adipocytes treated with vehicle or EGCG for 8 hr. Autophagy inhibitors (chloroquine (CQ), and 3-MA) were pretreated 30 min before EGCG or vehicle treatment. (n = 3 per condition, *<0.05, **p<0.01 compared to Vehicle control, #p<0.05 compared to EGCG controls)
